# Absence of Hydrochloric Acid in Cancer of the Stomach

**Published:** 1899-03-18

**Authors:** 


### ABSENCE OF HYDROCHLORIC ACID IN CANCER OF THE STOMACH.

In a clinical lecture on Carcinoma of the Stomach, recently delivered at Guy's Hospital, Dr. Hale White pointed out the importance of examining for the presence or absence of free hydrochloric acid in that viscus. There are several ways of testing for it; I always use, he said, a mixture of phloroglucin 2, and vanillin 1, in absolute alcohol 100 parts. Having this solution ready prepared you give the patient a test meal, that is to say, some simple food that will excite the gastric secretion. A very good meal for that purpose is a roll, a glass of milk, and the white of one or two eggs, either cooked or uncooked. Some people give a little beef steak and a roll, and a glass of milk. Then an hour and a half or two hours afterwards you can easily syphon out the contents of the stomach, filter them, and, in an evaporating dish, mix with the filtrate a few drops of your solution already prepared. Warm the mixture, and if hydrochloric acid is present, as it evaporates it turns a most brilliant crimson. The interest of this test is this: if you take people not suffering from carcinoma of the stomach and give them a test meal, nearly all will show some hydrochloric acid, while if you apply a like test in the case of patients with cancer of the stomach, by far the greater number will not show any, so that if hydrochloric acid be not present it is very strong presumptive evidence that the patient is suffering from carcinoma of the stomach. Dr. Hale White went on to say that he had only once known a case with gastric carcinoma in which this test showed distinctly that there was hydrochloric acid in the contents of the stomach but he said, that why this acid should be so rarely found in such cases was not known.

